# 1348. A Novel Host-Protein Signature Comprising TRAIL, IP-10 and CRP Differentiates Bacterial from Viral Infection in COPD Patients with Suspected Lower Respiratory Tract Infection

**DOI:** 10.1093/ofid/ofab466.1540

**Published:** 2021-12-04

**Authors:** Salim Halabi, Shachaf Shiber, Michal Stein, Meital Paz, Tanya Gottlieb, Eran Barash, Roy Navon, Einat Moscoviz, Tahel Ilan Ber, Liran Shani, Neta Petersiel, Olga Boico, Mordechai Grupper, Einav Simon, Dani Kirshner, Noa Avni, Daniel Haber, Yasmin Maor, Claire S Guetta, Ynon Lishtzinsky, Shirly Yanai, Michael Drescher, Kfir Oved, Eran Eden, Ami Neuberger, Mical Paul

**Affiliations:** 1 Carmel medical center, Haifa, HaZafon, Israel; 2 Rabin Medical Center, Petah Tikva, HaMerkaz, Israel; 3 MeMed Diagnostics, Haifa, HaZafon, Israel; 4 MeMed, Haifa, Israel, Haifa, Hefa, Israel; 5 MeMed Diagnostics Ltd, Tirat Carmel, Hefa, Israel; 6 Rambam Health Care Campus, Haifa, HaZafon, Israel; 7 Rambam Medical Center, Haifa, HaZafon, Israel; 8 Wolfson Medical Center and Tel Aviv Univversity, Holon, HaMerkaz, Israel; 9 Rabin medical center, Tel Aviv, HaMerkaz, Israel; 10 MeMed, Haifa, HaZafon, Israel

## Abstract

**Background:**

Identifying infectious etiology is often challenging, yet essential for patient management, including antibiotic use. Studies have shown that a host signature comprising TNF-related apoptosis induced ligand (TRAIL), interferon gamma induced protein-10 (IP-10) and C-reactive protein (CRP) accurately differentiates bacterial from viral infection with negative predictive value >98%. Performance data was lacking in chronic obstructive pulmonary disease (COPD) patients with suspected lower respiratory tract infection (LRTI).

**Methods:**

Adults aged >18 years with suspected LRTI were prospectively recruited at 3 medical centers (OBSERVER; grant #684589; NCT003011515). Reference standard infection etiology was adjudicated by 3 independent experts based on clinical, laboratory, microbiological, radiological and follow-up data. Host signature generates a bacterial likelihood score (0-100), providing three results: viral (0-35), equivocal (35-65) and bacterial (65-100). Experts were blinded to the signature result.

**Results:**

Out of 583 adults recruited with suspected LRTI, 422 met infectious criteria, of whom 48 had a recorded history of COPD. 19 cases were adjudicated by the experts as bacterial, 14 as viral and 15 were indeterminate (Figure 1). The mean age was 68.2 years (standard deviation 12.3); 33 (68.8%) presented after two or more days of symptoms and 38 (79.2%) were hospitalized for a median of 6 days. 15 (31.2%) were female. For the patients adjudicated bacterial or viral labels (n=33), the discharge diagnoses were: COPD exacerbation, 12 cases (36.4%); pneumonia, 12 cases (36.4%) (3.0%); acute bronchitis, 2 cases (6.1%); upper RTI ,1 case; unspecified viral infection 1 case (3.0%); or other, 5 cases (15.2%). Host signature correctly classified all 19 bacterial cases and 8 of the viral cases, providing accurate etiology labels for 27/33 COPD patients with reference standard labels (81.8%). The remaining 6 viral cases received equivocal scores (18.2%).

COPD patient enrollment and etiology labels in the Observer study

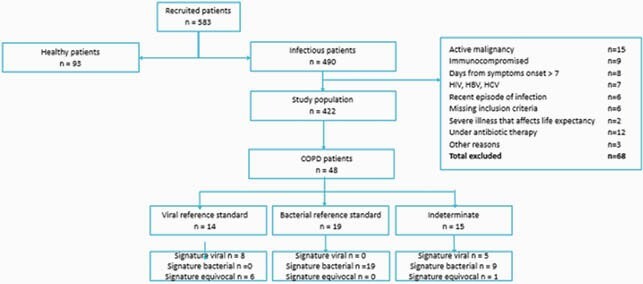

**Conclusion:**

Host signature accurately differentiates between bacterial and viral infections in patients with COPD history, supporting potential to improve management among these patients frequently admitted for RTIs.

**Disclosures:**

**Michal Stein**, **MeMed** (Employee) **Meital Paz, MD**, **MeMed** (Employee) **Tanya Gottlieb, PhD**, **MeMed** (Employee, Shareholder) **Eran Barash, MA**, **MeMed** (Employee) **Roy Navon, MSc**, **MeMed** (Employee, Shareholder) **Einat Moscoviz, BSc+ MBA**, **MeMed** (Employee) **Tahel Ilan Ber, MD**, **MeMed** (Employee, Shareholder) **Liran Shani, MD**, **MeMed** (Employee) **Olga Boico, PhD**, **MeMed** (Employee) **Einav Simon, PhD**, **MeMed** (Employee, Shareholder) **Noa Avni, PhD**, **MeMed** (Employee) **Kfir Oved, MD, PhD**, **MeMed** (Board Member, Employee, Shareholder) **Eran Eden, PhD**, **MeMed** (Board Member, Employee, Shareholder)

